# Defect Characterization of the SiO_2_/Si Interface Investigated by Drift-Assisted Positron Annihilation Lifetime Spectroscopy

**DOI:** 10.3390/nano16030156

**Published:** 2026-01-23

**Authors:** Ricardo Helm, Werner Egger, Catherine Corbel, Peter Sperr, Maik Butterling, Andreas Wagner, Maciej Oskar Liedke, Eric Hirschmann, Johannes Mitteneder, Michael Mayerhofer, Kangho Lee, Georg S. Duesberg, Günther Dollinger, Marcel Dickmann

**Affiliations:** 1 Institute for Applied Physics and Measurement Technology, University of the Bundeswehr Munich, 85579 Munich, Germany; werner.egger@unibw.de (W.E.); peter.sperr@unibw.de (P.S.); johannes.mitteneder@unibw.de (J.M.); michael.mayerhofer@unibw.de (M.M.); guenther.dollinger@unibw.de (G.D.); marcel.dickmann@unibw.de (M.D.); 2 LSI, CEA/DRF/IRAMIS, CNRS, Ecole Polytechnique, Institut Polytechnique de Paris, 91120 Palaiseau, France; catherine.corbel@polytechnique.edu; 3 Reactor Institute Delft, Delft University of Technology, 2629 JB Delft, The Netherlands; m.butterling-2@tudelft.nl; 4 Institute of Radiation Physics, Helmholtz Center Dresden-Rossendorf, 01328 Dresden, Germany; a.wagner@hzdr.de (A.W.); m.liedke@hzdr.de (M.O.L.); e.hirschmann@hzdr.de (E.H.); 5 Institute of Physics, University of the Bundeswehr Munich, 85579 Munich, Germany; kay.lee@unibw.de (K.L.); georg.duesberg@unibw.de (G.S.D.)

**Keywords:** ultra thin-films, interface defects, drift-assisted positron annihilation lifetime spectroscopy, oxide–semiconductor interface

## Abstract

This study demonstrates drift-assisted positron annihilation lifetime spectroscopy on a p-type (100) silicon substrate in a MOS capacitor, using an applied electric field to control the spatial positron distribution prior to annihilation. The device was operated under accumulation, depletion, and inversion conditions, revealing that the internal electric field can drift-transport positrons either toward or away from the SiO_2_/Si interface, acting as a diffusion barrier or support, respectively. Key positron drift-transport parameters were derived from lifetime data, and the influence of the non-linear electric field on positron trapping was analyzed. The comparison of the presented results to our previous oxide-side drift experiment on the same metal-oxide–silicon capacitor indicates that the interface exhibits two distinct sides, with different types of defects: void-like and vacancy-like ($P_{b}$ centers). The positron data also suggest that the charge state of the $P_{b}$ centers likely varies with the operation mode of the MOS, which affects their positron trapping behavior.

## 1. Introduction

With the rise of artificial intelligence, quantum computing, and other advanced data processing and sensor systems, the demand for high-quality and increasingly efficient electronic devices is steadily growing across many technology sectors. Achieving higher performance requires increasing transistor density, which involves shrinking the size of individual transistors [1]. This miniaturization necessitates a shift away from conventional silicon-based metal-oxide–semiconductor (MOS) structures to new materials and transistor concepts, such as thin-film transistors (TFTs) [2], 2D integrated devices [3], and 3D integrated devices like gate-all-around field-effect transistors (GAAFETs) [1]. These approaches introduce new material systems that incorporate advanced materials, such as transition metal dichalcogenides on high-k dielectrics [4,5]. However, as device dimensions decrease, the impact of interface defects—which already limits performance in conventional devices—becomes even more significant [6]. Minimizing these defects is crucial to maintaining high performance and device reliability. To effectively reduce interface defects, proper identification is necessary. Developing advanced characterization techniques for these new material systems and their reduced dimensions is essential for building a solid foundation to understand their interfacial interactions.

Low-energy monochromatic positron beams of variable energy, continuous and pulsed, are a powerful tool for probing atomistic defects in layered systems [7,8,9], including dielectric semiconductor structures [10,11]. However, due to the implantation profile of slow positrons, the detection of buried ultra-thin layers—such as layers and interfaces thinner than 10 nm—remains challenging. This limitation can be addressed by combining pulsed-beam positron annihilation lifetime spectroscopy (PALS) with an applied sample bias, which creates strong electric fields that transport positrons toward specific regions before annihilation. Previous studies have demonstrated the successful positron transport in MOS capacitors using continuous-beam Doppler broadening spectroscopy (DBS) [12,13,14]. More recently, we reported drift-assisted PALS measurements in a 180 nm SiO_2_/Si layer, revealing how internal electric fields affect positron annihilation and positronium formation and providing insights into the microstructure of the oxide and the SiO_2_/Si interface [15].

In this work, we extend this approach by performing the first beam-based, drift-assisted PALS experiment directly in the silicon substrate of a MOS capacitor, operated under accumulation, depletion, and inversion. By exploiting the depletion zone formed in the near-interface region of the silicon substrate, we control the positron annihilation location via the gate voltage of the MOS capacitor. In addition to the limitation of the width of the positron implantation profile, positron beams pose several other limitation that needs to be kept in mind. While this method is a unique way to characterize atomistic defects, it yields defect information down to a defect concentration of 1 × 10^−7^. Further, typical positron implantation energies in pulsed positron beams range from 0.5–20 keV, which translates to an implantation depth of several nanometers to 100 nm for high density metals and 100 nm–100μm for low density materials, like polymers.

When the MOS capacitor is operated in depletion or inversion, annihilation at the interface can be suppressed by drifting positrons into the silicon bulk. Conversely, under accumulation, positrons are collected at the SiO_2_/Si interface, producing a distinct annihilation signal that can be attributed to defects located near or directly at the SiO_2_/Si interface.

## 2. Materials and Methods

### 2.1. Metal-Oxide–Silicon System

The investigated Metal-Oxide–Silicon (MOS) capacitor was fabricated from a commercial-grade p-type silicon(100) substrate. A thermal wet oxidation process was used to grow an oxide layer with a thickness of 180(10) nm on the substrate surface. To allow the application of a bias voltage, a 40(10) nm thick aluminum gate electrode was deposited onto the oxide by vapor deposition. The backside of the wafer was coated with a thin aluminum layer to ensure good electrical contact [15]. The positrons are implanted perpendicularly through the gate electrode and oxide layer, corresponding to a total thickness of approximately 220 nm, before reaching the underlying silicon substrate.

With the analysis of the high-frequency capacitance and conductance as a function of gate voltage, key electrical parameters of the MOS capacitor were determined. A voltage range of −8 to +7 V was used for the voltage sweep. The mismatch between the work function of the aluminum gate and the silicon substrate, together with the presence of interface traps at the SiO_2_/Si interface, results in a potential shift known as the flatband voltage. Following the methodology described in [16], the flatband voltage was found to be $U_{\mathrm{fb}}=-0.9\;V$.

**Figure 1:**
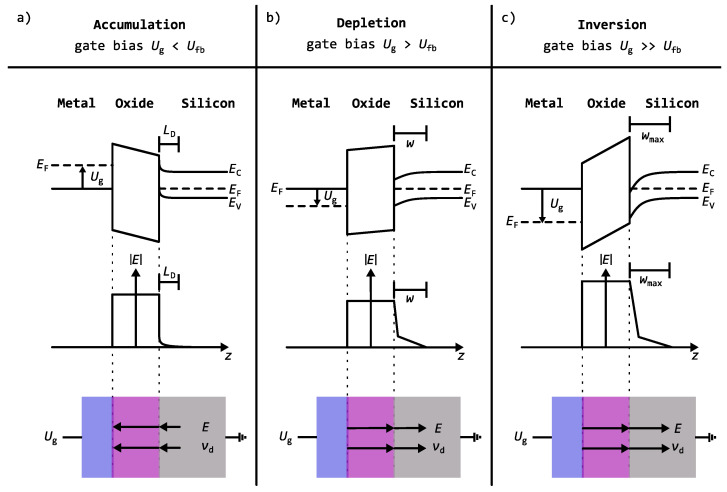
Schematic representation of the band diagram, the electric field distribution in *z*, the electric field direction, and the positron drift direction for a MOS capacitor operating in (**a**) accumulation, (**b**) depletion, and (**c**) inversion modes. Different operation modes result in different field distributions in the silicon substrate. $U_{g}$ is the applied gate voltage, $U_{\mathrm{fb}}$ the flat-band voltage, $E_{F}$ the Fermi energy, $E_{C}$ the conduction band energy, $E_{V}$ the valence band energy, *E* the electric field, $v_{d}$ the drift velocity of the positron, $L_{D}$ the Debye length and *w* the width of the depletion layer.

At flatband ($U_{g}=U_{\mathrm{fb}}$), the band bending of the valence and conduction bands near the silicon interface vanishes (see Figure 1); therefore, no electric field is present in either the SiO_2_ or the silicon substrate.

The interface trap density $D_{\mathrm{it}}$ was estimated using the conductance method described in [17,18]. This analysis yields an interface trap density of $D_{\mathrm{it}}=10^{10}$–$10^{11}\;eV^{-1}cm^{-2}$. Such a low trap density indicates a high-quality oxide–semiconductor interface, even in the absence of post-oxidation annealing [19,20].

Finally, the bulk acceptor concentration was extracted from the C-V measurements, resulting in $N_{A}=1.20\left(9\right)\times 10^{15}\;cm^{-3}$.

### 2.2. MOS Operational Modes

The drift behavior in the near-surface region of the buried silicon layer is complex because both the width of this region and the electric field profile strongly depend on the applied gate voltage. By varying the gate voltage, the degree of band bending at the oxide/silicon interface can be precisely controlled. Depending on the applied bias, the MOS capacitor operates in one of three regimes—accumulation, depletion, and inversion—each characterized by a specific surface potential at the oxide interface. These regimes, in turn, define the direction, strength, and spatial extent of the electric field within the silicon substrate.

Figure 1a–c illustrates the three operating modes of a p-type MOS capacitor and their correlation with the gate voltage. The top panel shows the corresponding band diagrams and the band bending near the semiconductor surface resulting from the surface potential in each mode. The middle panel depicts the electric field distribution, while the bottom panel shows the direction of the electric field and the resulting positron drift velocity induced by it:

(a) Accumulation mode ($U_{g}<U_{\mathrm{fb}}$): In this regime, the electric field within the substrate is primarily determined by the Debye screening length $L_{D}$ of the majority carriers. From the Poisson equation, the thickness of the accumulation layer can be estimated as $\pi L_{D}/\sqrt{2}\approx 260\;nm$ at 300 K and a doping concentration of $1.2\times 10^{15}\;cm^{-3}$ [21]. The electric field is directed toward the oxide–silicon interface, resulting in a positron drift from the silicon substrate toward the interface.

(b) Depletion mode ($U_{g}>U_{\mathrm{fb}}$): When the gate voltage exceeds the flatband voltage, majority carriers are repelled, creating a depletion zone. The width *w* of this zone increases with higher gate voltages and extends deeper into the substrate until an inversion channel begins to form.

(c) Inversion mode ($U_{g}>>U_{\mathrm{fb}}$): At sufficiently high gate voltages, the near-surface region becomes electrically inverted, and minority carriers accumulate at the interface. The maximum depletion width $w_{\max}$, determined from the minimum and maximum capacitances in the C-V measurements, is found to be 618(62) nm. This agrees well with the theoretical value of 700 nm for our doping concentration [21,22]. Within this region, the electric field decreases approximately linearly, whereas beyond $w_{\max}$, it decays exponentially. In both depletion and inversion mode, the electric field is directed toward the bulk of the silicon substrate.

### 2.3. Positron Annihilation Lifetime Spectroscopy

The low-energy Positron-Annihilation Lifetime Spectroscopy (PALS) with in-situ gate voltage variation was performed at the Mono-energetic Positron Source (MePS) at the Helmholtz Center Dresden-Rossendorf [23].

**Figure 2:**
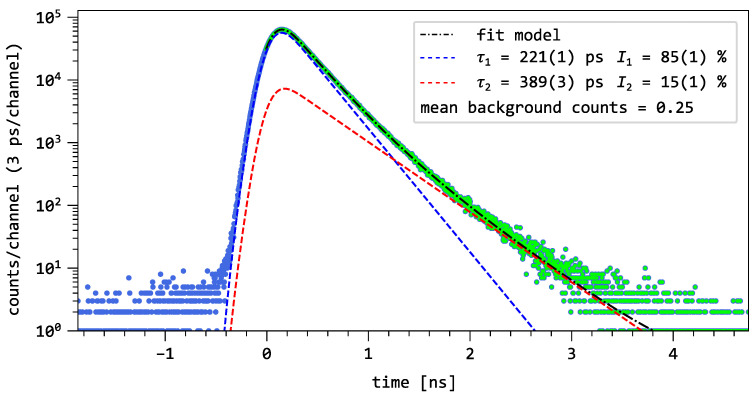
Positron decay spectrum of p-type Silicon at 8 keV measured with the MePS instrument. The blue markers are the measured data points, and the green markers are the data points used for the fit model. The colored dashed line corresponds to the decay components delivered by the model, and the black dash-dotted line corresponds to the fit model.

Positron lifetime spectra were recorded as a function of the implantation energy ranging from 1 to 11 keV at voltages between −30 V and +30 V applied to the MOS capacitor. The gate bias was supplied by an Iseg DPS High Precision HV module from Iseg Leistungselektronik GmbH, 01454 Radeberg, Germany with an uncertainty of 0.5 V. The voltage $U_{g}$ was applied between the Al gate at the top of the SiO_2_ layer and the ohmic Al contact at the back of the Si substrate, which was kept at ground potential. Each positron lifetime spectrum was recorded with at least $1\times 10^{7}$ counts and the peak-to-background ratio was higher than $1\times 10^{6}$:1 (see Figure 2). The total time resolution of MePS, including the pulsing system and detector, was 230 ps (FWHM) throughout the experiment. The time resolution was determined by characterizing a well-known reference material, yttrium-stabilized zirconia, and was stable for all implantation energies. A three-component decomposition was employed to fit all lifetime spectra with three decay components, achieving high accuracy with a reduced $\chi^{2}\leq 1.20$ for the unconstrained fits. The starting values and channel ranges were kept constant for all spectra, ensuring consistent analysis conditions.

For the reference measurement of the pure silicon substrate, a conventional PALS set-up was used, similar to the one described in [24]. The conventional spectrometer uses a semi-digital setup incorporating a DRS4 Evaluation Board and an analogue circuit as an external trigger source. The time resolution of the conventional setup is in the range of 250 ps with a peak-to-background ratio of $1\times 10^{4}$:1.

## 3. Results and Discussion

### 3.1. Bare Silicon Substrate

#### 3.1.1. Bulk Lifetime

Figure 2 presents the positron annihilation lifetime spectrum of the untreated p-type silicon wafer at an implantation energy of 8 keV. The spectrum is a decay spectrum in time. The blue points correspond to the measured data, while the green points represent the data used for the fit model. In the case of the spectrum in Figure 2, the data are modeled as

(1) $$D_{\exp}\left(t\right)=\left[IRF*\left(\frac{I_{1}}{\tau_{1}}e^{-t/\tau_{1}}+\frac{I_{2}}{\tau_{2}}e^{-t/\tau_{2}}\right)\right]+B$$

where $D_{\exp}$ is the experimental data, IRF is the instrument resolution function and *B* is the background of the spectrum. $I_{1}$ and $I_{2}$ are the relative intensities, and $\tau_{1}$ and $\tau_{2}$ are the lifetimes of components 1 and 2 respectively. The latter parameters are obtained by fitting the model to the data [25]. In the logarithmic representation of the decay spectrum, two distinct decay regions can be identified and extracted from the spectrum by the fit component as indicated by the coloured dashed lines. The values of the model components, along with their respective statistical uncertainties, are provided in the legend.

The measurement of the untreated p-type silicon wafer with MePS yielded two lifetime components: a short component of $\tau_{1}=221\left(1\right)\;ps$ and a longer component of $\tau_{2}=389\left(3\right)\;ps$. To verify the value of the first component, a reference measurement was carried out using a conventional laboratory setup with a ^22^Na source, as described in Section 2.3.

The reference spectrum was analyzed using a three-component decomposition. The source contribution—annihilation of positrons in the ^22^Na salt and the Kapton cladding—consists of a component with a lifetime of approximately 398 ps and intensity of 10%, along with a longer component with a lifetime of around 2–3 ps and an intensity of 1.5%. Among the three components, only one can be attributed to positron annihilation in the defect-free bulk. The shortest lifetime of 221(2) ps was identified as the bulk lifetime of the silicon substrate. This lifetime result agrees with the beam-based measurement and with previous measurements on various silicon materials [26,27,28]. We will refer to this lifetime value as the bulk lifetime $\tau_{b}$ of defect-free silicon.

#### 3.1.2. Surface States and Diffusion to the Surface

The implantation profile P(z,E) describes the linear density distribution of thermalized positrons before diffusion or electric-field-assisted drift. It depends on both the material density and the incident positron energy. In the MOS capacitor under an applied gate voltage $U_{g}$, this profile is additionally shaped by the layered Al/${\mathrm{SiO}}_{2}$/Si structure. By varying $U_{g}$, the drift of positrons in ${\mathrm{SiO}}_{2}$ and Si can be controlled, thereby influencing the annihilation probability at the interfaces and the accessible positron quantum states in the bulk materials. The Al surface, the Al/${\mathrm{SiO}}_{2}$ interface, and the ${\mathrm{SiO}}_{2}$/Si interface can each host distinct annihilation states, with the ${\mathrm{SiO}}_{2}$/Si interface contributing significantly to the observed lifetime spectrum through diffusion, internal electric fields, or gate-voltage-controlled drift.

In slow-positron beam experiments, diffusion is a critical factor, particularly at low implantation energies (0.5–4keV) and in inhomogeneous systems such as thin films or multilayer structures, where the effective positron diffusion length $l_{+}$ is comparable to the layer thickness or the distance to trapping centers [29]. Under these conditions, the annihilation process must be described using the diffusion-trapping model [30,31,32].

**Figure 3:**
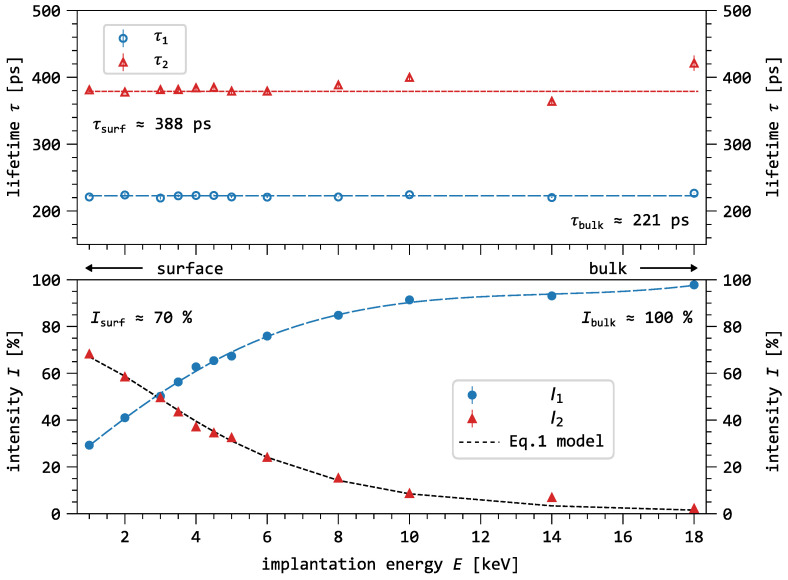
Two-component decomposition of the bare silicon sample measured at the pulsed positron beam facility (MePS) as a function of positron implantation energy. Both lifetime components remain essentially constant over the investigated energy range. However, the intensity gradually transitions from one component to the other with increasing implantation energy, which can be attributed to positron back-diffusion towards the surface. Positrons that diffuse back to the surface may become trapped in surface defects, exhibiting a characteristic lifetime of approximately 360–500 ps [32,33,34,35].

Figure 3 shows the two-component decomposition of the bare silicon spectra measured as a function of implantation energy. The first component exhibits a lifetime with a mean value of 222(2) ps, with an energy-dependent intensity ranging from 29% to 98%. The second component shows a lifetime with a mean value of 385(5) ps, and its intensity varies with energy from 68% to 2%. At low positron implantation energies (1–3 keV) an additional long component with a lifetime of 2–3 ns and a low intensity of ≈3% can be observed.

The reduction of $I_{2}$ and $I_{3}$ with increasing implantation energy suggests that $\tau_{2}$ and $\tau_{3}$ originate either from defects close to the surface, or from the native oxide layer on the silicon surface. Indeed, a lifetime of 360–500 ps is typical for a positron annihilation state at surfaces [33,34,35]. At medium implantation energies (3–5 keV), the surface component still contributes 50–30% to the average lifetime, indicating that a fraction of positrons are still reaching the silicon surface.

By applying the diffusion model developed by G. Kögel [32] to various observables of the lifetime measurement, the effective positron diffusion length can be determined. This can be achieved by fitting the intensity model to the surface intensity $I_{2}$. The surface intensity originates from the positrons annihilating in surface states. $I_{2}$ decreases with increasing implantation energy, which is mainly dependent on the diffusion properties of the positrons in the material. The model is expressed as

(2) $$I_{2}=\frac{\nu \left(1-p\sqrt{\frac{\pi }{2}}\exp\left(\frac{p^{2}}{4}\right)\mathrm{erfc}\left(\frac{p}{2}\right)\right)}{\left(1+\sqrt{D_{+}\left(\lambda_{b}-\lambda_{s}\right)}\right)}$$

$$p=14\left[cm\right]\sqrt{\frac{\lambda_{b}-\lambda_{\mathrm{surf}}}{D_{+}}}{\left(\frac{E_{\mathrm{imp}}}{\left[\mathrm{keV}\right]}\right)}^{1.7}$$

where ν is the surface capture rate, $\lambda_{b}=\tau_{b}^{-1}$ is the annihilation rate in the bulk, $\lambda_{\mathrm{surf}}=\tau_{s}^{-1}$ is the annihilation rate at the surface, $D_{+}$ is the diffusion coefficient of positrons in the material, and $E_{\mathrm{imp}}$ is the implantation energy. The open parameters are ν and $D_{+}$.

The intensity $I_{2}$ as a function of the implantation energy is shown in Figure 3, where the dashed line in Figure 3 represents the intensity model from Equation (2). This model yields a positron diffusion constant of $D_{+}=0.88\left(9\right)cm^{2}s^{-1}$. However, it should be noted that surface charges induce band bending near the silicon surface, creating an intrinsic electric field that modifies the observed diffusion constant [36]. Further, we calculate the effective positron diffusion length $l^{+}=\sqrt{D_{+}\tau_{b}}$ to be 139(14) nm. Both parameters match previously reported values for p-type silicon with comparable doping concentrations [33,35].

According to the Einstein relation, the positron mobility can be expressed as

(3) $$\mu_{+}=\frac{eD_{+}}{k_{b}T}$$

With $D_{+}$ we can calculate the positron mobility ($\mu_{+}$) in the silicon substrate. Using Equation (3) and assuming a temperature of T=300 K, we obtain $\mu_{+}=35\left(3\right)cm^{2}V^{-1}s^{-1}$.

From the positron mobility, additional parameters can be derived to provide insights into the average behavior of positrons influenced by the electric field. For defect-free materials with a known bulk lifetime, we can define the drift distance $l_{d}=\mu_{+}E\left(r\right)\tau_{b}$ covered during its lifetime.

When an electric field is present, the post-thermalization motion of positrons is governed by the diffusion–drift equation [31]. In this framework, positron transport is described as the superposition of stochastic diffusion, drift by the electric field, and the defect trapping mechanism.

### 3.2. Field and Transport Modelling

**Figure 4:**
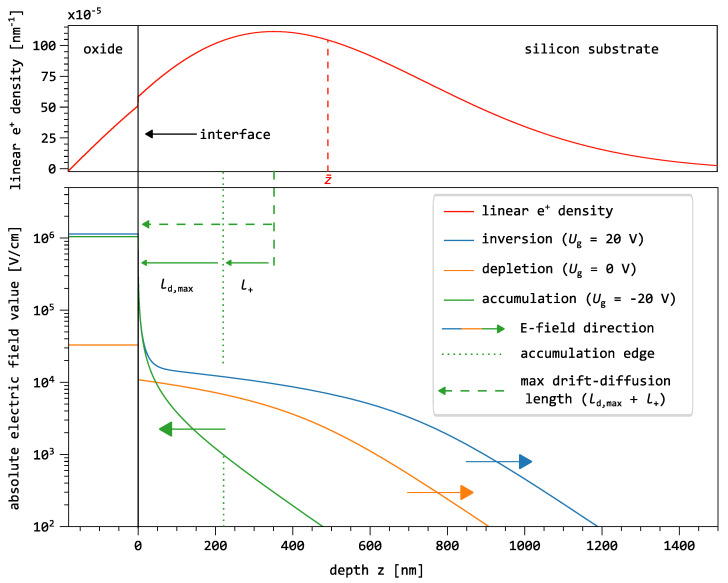
The top graph shows the positron implantation profile as a function of depth for an implantation energy of 11 keV, with $\bar{z}$ denoting the mean implantation depth. The total fraction of positrons implanted into the oxide is approximately 8%, while about 92% are implanted into the silicon substrate. The bottom graph presents the simulated absolute electric field values in the oxide and silicon substrate as a function of depth for the different operating modes of the MOS capacitor. The direction of the electric field at various gate voltages is indicated by arrows. The dashed green line denotes the maximum distance from which a positron can still reach the interface under combined drift–diffusion transport for a mobility $\mu_{+}=35\;cm^{2}V^{-1}s^{-1}$ and a diffusion length in silicon of 141(22) nm.

In monoenergetic slow-positron beam experiments, the positron implantation energy determines the probing depth within the material. The stopping profile of positrons, typically described by a Makhovian distribution, extends from the surface of the layered system to a depth primarily governed by the implantation energy and the material density. An example of such a one-dimensional positron distribution is shown in Figure 4. The upper graph displays the implantation profile—known as the Makhov profile [37]—for a positron energy of 11 keV along the *z*-direction. This represents the spatial distribution of thermalized positrons before diffusion and drift occur. For simplicity, and due to the low probability (≤1%) of positrons thermalizing within the aluminum gate at 11 keV, the thickness of the aluminum gate is neglected. The step observed in the one-dimensional positron distribution at the oxide–silicon interface arises from the difference in density and mean atomic number between the materials on either side of the interface.

Numerical simulations of the electric field distribution in the near-surface region of the silicon substrate were performed to aid the interpretation of the positron drift experiments. These simulations can be seen at the bottom of Figure 4 and were carried out using MOScap (version 1.8) [38], which is based on the PADRE device simulation code [39]. The modeled MOS structure consists of an idealized, infinitely thin aluminum gate, a 180 nm SiO_2_ layer, and a 5 μm p-type silicon substrate. The doping concentration of the p-Si substrate was chosen to match the experimentally determined value of $1.20\times 10^{15}\;cm^{-3}$. The work function difference between the gate and the substrate results in ΔΦ=−0.7 eV. As the model does not include fixed oxide charges, interface traps, or other non-idealities that can influence the flatband voltage, the flatband voltage is given by $U_{\mathrm{fb}}=\Delta \Phi /q$. Electric field and carrier distributions were calculated for three different operative conditions, i.e., gate voltages of −20 V (accumulation), 0 V (depletion), and +20 V (inversion). These voltages are well beyond the respective threshold conditions to drive the MOS in accumulation or inversion, respectively. This ensures that the maximum extent of the space-charge region and the corresponding electric field distribution within the silicon substrate are fully developed.

The lower graph in Figure 4 shows the simulated electric field distribution as a function of depth *z* for the accumulation, depletion, and inversion modes, with the field directions indicated by arrows. The electric field in the oxide is also depicted, along with the direction of the electric field in each operational mode.

For calculating the drift velocity, we estimate the mean electric field in the interface region of the silicon substrate. The surface field in silicon is related to the field in the oxide by

(4) $$E_{s}=\frac{\epsilon_{\mathrm{ox}}E_{\mathrm{ox}}}{\epsilon_{\mathrm{si}}}$$

In accumulation, we approximate the mean electric field as $E_{s}/e$, while in depletion and inversion we use $E_{s}/2$. For the maximum applied gate voltage of ±20 V this results in mean electric field values of approximately $1.3\times 10^{5}\;Vcm^{-1}$ in accumulation and $1.9\times 10^{5}\;Vcm^{-1}$ in inversion.

To get an idea of how much of the positron implantation density overlaps with the electric field distribution. The edge of the accumulation region marks the maximum distance from which a positron can still reach the interface under ideal drift. Further, the drift-diffusion length accounts for both the drift and diffusion processes, providing a rough estimate of the maximum distance from which positrons can still reach the interface in their lifetime.

In accumulation mode ($U_{g}<U_{\mathrm{fb}}$), the electric field is directed toward the SiO_2_/Si interface. The maximum drift distance that positrons can travel toward the interface within an exponential electric field can be estimated as

(5) $$l_{\mathrm{dm}}=L\ln\left(1+v_{d}\tau_{b}/L\right)$$

where *L* represents the characteristic length of the field decay, here approximated as 40 nm, corresponding to the transition region from the surface field to the exponentially decaying field in Figure 4. The term $v_{d}$ denotes the drift velocity in the mean electric field, and $\tau_{b}$ is the positron lifetime in defect-free silicon.

Using the mobility and bulk lifetime values derived in the previous chapter, and an electric field of $\langle E\rangle =1.3\times 10^{5}\;Vcm^{-1}$ (for $U_{g}=-20\;V$), the maximum drift distance is estimated to be about 220 nm, indicated by the dotted vertical line (the accumulation edge) in Figure 4.

To also account for diffusion, which contributes additively to the transport process, a combined drift–diffusion distance can be estimated as the sum of the maximum drift length $l_{\mathrm{dm}}$ and the effective positron diffusion length $l^{+}$. This yields a total transport range of approximately 360 nm, marked by the dashed vertical line in Figure 4. Positrons implanted beyond this distance are unlikely to reach the interface before annihilation.

### 3.3. Buried Substrate Under Bias Condition

By applying a gate voltage, the electric field in the near-surface region of the buried silicon can be tuned, modifying the drift that adds to diffusion. This makes it possible to either enhance or suppress positron transport to the interface. To demonstrate this, we measured the MOS capacitor at a fixed implantation energy of 11 keV while varying the gate voltage from −30 V to 30 V.

**Figure 5:**
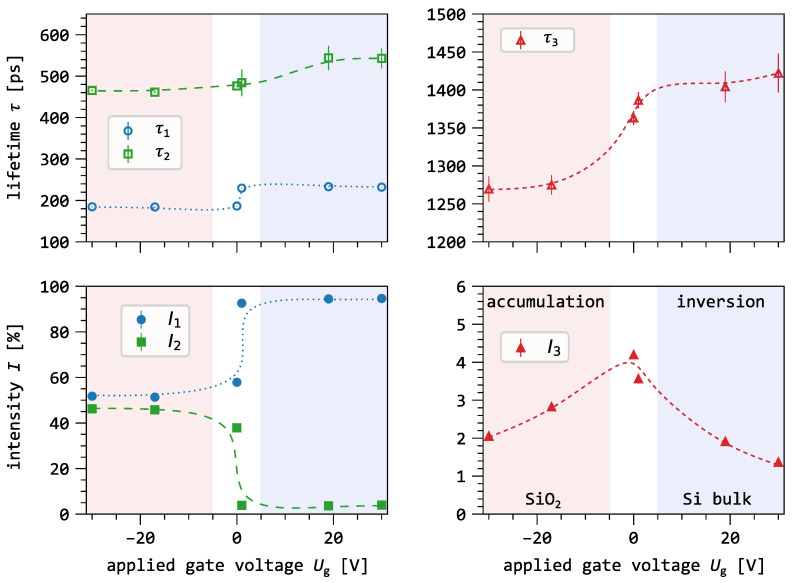
Three-component decomposition of the lifetime spectra at an implantation energy of 11 keV as a function of the gate voltage $U_{g}$. The red and blue shaded regions indicate the general direction of the electric field: toward the oxide/substrate interface in the red region and the substrate bulk in the blue region. The lines connecting the data points are only to guide the eye.

Figure 5 shows the results of the three-component lifetime spectra decomposition as a function of gate voltage for a positron implantation energy of 11 keV. At this implantation energy, approximately 92% of the positrons are implanted into the silicon substrate, while the remaining 8% stop within the Al gate and the SiO_2_ layer. The red and blue shaded areas in Figure 5 correspond to the operating regimes of the MOS capacitor and thus indicate the direction of the electric field (see Figure 1). In the red region (accumulation mode), the electric field drives positrons toward the SiO_2_/Si interface. In the blue region (inversion mode), the drift is directed toward the silicon bulk. The intermediate white region marks the transition between these two regimes, where the dominant drift direction changes from the interface to the bulk.

The lifetime of the first component $\tau_{1}$ exhibits a mean value of 185(2) ps in accumulation mode (see Figure 1). When transitioning to depletion/inversion mode, this mean value sharply increases to 232(2) ps. The corresponding intensity $I_{1}$ rises from 52% in accumulation to 95% in inversion, with a sudden increase observed between 0 V and 1 V. The lifetime of the second component $\tau_{2}$ increases from 467(4) ps in accumulation mode to 524(49) ps in inversion mode, with a noticeable jump at $U_{g}\approx 1\;V$. The intensity $I_{2}$ decreases sharply from 46% in accumulation to 4% in inversion, highlighting a rapid decrease in the depletion region. The lifetime of the third component ($\tau_{3}$) reaches a minimum of 1270(16) ps in accumulation mode and a maximum of 1422(26) ps in inversion mode. The intensity $I_{3}$ displays a symmetric dependence around 0 V, decreasing in both accumulation and inversion mode. The maximum intensity observed is 4%. Table 1 summarizes the results of the three-component lifetime analysis.

Additionally, we evaluated the lifetime spectrum at 1 keV, where all positrons thermalize within the aluminum gate, to assess the possibility of ballistic positron transport from the silicon substrate across the oxide layer into the gate material, as previously observed for much thinner oxides [13]. The 1 keV-spectrum reveals a dominant lifetime of 265(2) ps with an intensity of 64%, and two surface components $\tau_{2}=454\left(4\right)\;ps$, $I_{2}$ = 35% and $\tau_{3}=2702\left(40\right)\;ps$ with 2% intensity. The shortest lifetime of 265(2) ps is significantly higher than the bulk aluminum lifetime of 163 ps. This value is characteristic of positron annihilation at monovacancies or divacancies in aluminum [40], indicating that the evaporated aluminum gate is highly defective and that positrons are fully trapped at vacancy-like defects within the gate material.

### 3.4. e^+^ Transport in the Silicon Substrate

In Figure 4, we present the simulated electric field in the near-surface region of the buried silicon, alongside the calculated one-dimensional positron density distribution after thermalization from 11 keV implantation. Based on these simulations, we have also determined key transport parameters, such as the effective positron diffusion length $l_{+}$ and mobility $\mu_{+}$ within the buried silicon layer. In the following, we combine these results to analyze the transport behavior of positrons in the buried silicon and to demonstrate how the application of a gate voltage can be used to control their annihilation characteristics.

#### 3.4.1. e^+^ Drift in Inversion and Depletion Mode

In the depletion and inversion modes, the electric field points from the oxide/silicon interface toward the bulk of the substrate. This field adds a directed drift component to the otherwise random diffusion of positrons, producing a net current away from the interface. As a result, a strong diffusion barrier forms, which suppresses back-diffusion to the interface even at relatively low field strengths. Additionally, under depletion conditions with an average field of $\langle E\rangle =5.0\times 10^{-3}\;Vcm^{-1}$, the mean drift length of a positron before annihilation is about 390 nm, nearly two orders of magnitude larger than the width of the interfacial region. Consequently, even positrons implanted close (∼1 nm) to the interface are efficiently swept into the bulk before they can lose enough thermal energy to become trapped.

Under inversion conditions, the electric field extends mostly linearly across the depletion region, which reaches a maximum width of $w_{\max}$. Closer to the interface—within approximately 40 nm (see Figure 4)—the field strength increases sharply, reaching an average value of $\langle E\rangle =2.7\times 10^{5}\;V/cm$. This is about two orders of magnitude higher than the mean field in the depletion layer. Such a strong near-interface field effectively shields the interface and prevents most positrons from reaching it. As in the oxide, positron trapping in semiconductors occurs primarily via acoustic phonon scattering, which involves multiple successive collisions near a defect to disperse the binding energy of the defect [41,42]. Consequently, under the high field conditions, the probability of positron trapping at the interface is strongly reduced.

This behavior is clearly reflected in the 11 keV data shown in Figure 5. As the gate voltage increases from the transition region into positive values (blue region), the lifetime component $\tau_{1}$ rises sharply to values close to the bulk lifetime 221 ps, accompanied by an increase of its intensity $I_{1}$ to about 95%. At the same time, the intensity of the second component $I_{2}$ decreases and almost disappears.

The lifetime components $\tau_{2}$ and $\tau_{3}$ arise from pick-off annihilation of o-Ps, since they are above the low-density limit of 500 ps for free positrons [43]. Conversely, Figure 2 showed that o-Ps formation is not observed for defect-free silicon. Thus, the persistence of the second and third lifetime components, even at high implantation energies, is attributed to positrons that are still implanted into the preceding layers, resulting in annihilation within the aluminum gate or the SiO_2_ layer. The observed trends, namely, the convergence of $\tau_{1}$ to $\tau_{b}$, the increase in $I_{1}$, and the reduction of $I_{2}$, demonstrate that we successfully drifted the positrons away from the oxide/substrate interface and into the bulk of the substrate.

#### 3.4.2. e^+^ Drift in Accumulation Mode

At negative gate voltages, the electric field points toward the SiO_2_/Si interface, thus enhancing positron drift transport back to the interface. Assuming simple ballistic transport, the previously estimated thickness of the accumulation layer (260 nm) serves as an approximate range over which the field can influence positrons. When combined with the derived effective diffusion length in silicon, we estimate that positrons can reach the interface from depths up to 400 nm. However, the limited overlap between the positron implantation profile and this drift-diffusion range (Figure 4) indicates that fewer than half of the implanted positrons reach the interface before annihilation.

The C-V measurements show that accumulation is fully established below ≈−8 V, meaning the field-affected region does not grow with more negative voltages. Therefore, for both −17 V and −30 V, we assume that the same fraction of positrons is affected by the drift. This saturation effect is reflected in the results shown in Figure 5 at negative voltages.

The decrease in $\tau_{1}$ near the flatband condition cannot be explained by annihilation in the defect-free silicon alone. Based on earlier drift experiments [13,44,45], we consider the following possible mechanisms for the positron back-drift:

1. Transport to the gate over the oxide [13]: Previous studies have shown that positrons can cross the interface and annihilate in the gate material. However, in our measurements, no lifetime component around 265 ps, which would correspond to the annihilation signature of the Al gate, was detected. Such a signal would be expected with an intensity of approximately 25%, but none was observed.
2. Drift from the silicon into the oxide toward the Al/SiO_2_ interface [44]: Here, substrate-implanted positrons can surmount the interface and are subsequently affected by the oxide field, which drives them further toward the Al/SiO_2_ interface. If strong fields drove positrons from the substrate into the oxide, the lifetime spectra at 0 V (weak fields) and −30 V (strong fields) would differ. As no such behavior is observed, this transport pathway can be excluded.
3. Trapping at the SiO_2_ interface [45]: The interface acts as an efficient positron sink, and all positrons that reach the interface annihilate there. This provides the most consistent explanation for the observed lifetime behavior. As gate voltage becomes more negative, $I_{1}$ decreases sharply while $I_{2}$ increases, indicating enhanced trapping of positrons at interface defects. Since drift only affects part of the positrons implanted into the substrate, this results in a reduced bulk lifetime [25].

### 3.5. Defects at the Interface

**Figure 6:**
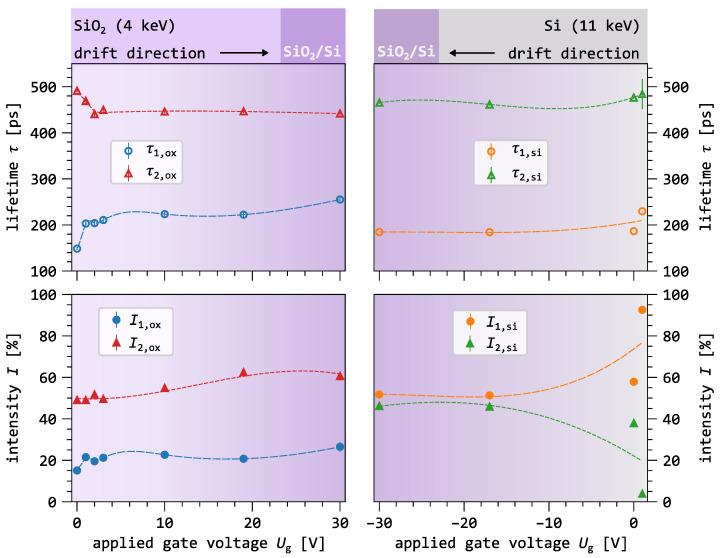
Comparison of lifetime components as a function of applied gate voltage. The left column shows results for positron drift from the oxide side toward the SiO_2_/Si interface, while the right column shows results for drift from the silicon side toward the same interface. A violet gradient schematically illustrates the interface influence on the positron lifetime, with darker shades indicating a larger fraction of annihilation events occurring closer to the interface.

Before analyzing the implications of the drift experiment for the microstructural properties of the SiO_2_/Si interface, we briefly review previously reported results on positron drift in the adjacent oxide layer [15]. Figure 6 compares positron lifetime measurements for drift toward the interface from the oxide side (left data set, taken from [15]) and from the silicon substrate side (right data set, shown in Figure 5).

In the oxide drift experiment [15], applying a gate voltage to the MOS capacitor suppresses positronium formation and enhances annihilation of free positrons within the oxide. These results confirm successful drift toward the SiO_2_/Si interface [15]. The electric field reduces positron trapping in the oxide by accelerating positrons between phonon interactions, thereby limiting energy loss and decreasing localization at the defect sites. As drift to the interface increases, the first lifetime component rises in both intensity and lifetime, reaching a maximum of $\tau_{1,\mathrm{ox}}=255\left(7\right)\;ps$ (Figure 6, left side). After correcting for para-positronium (p-Ps) contributions by fixing the p-Ps lifetime to the vacuum value of 125 ps and its intensity to $I_{o-\mathrm{Ps}}/4$, an additional unresolved lifetime component of 275(7) ps emerges, which is attributed to interface-related annihilation [15].

The interface region can be divided into two distinct transition layers. First, a structural transition layer arises from the relaxation and adaptation of stoichiometric SiO_2_ to the underlying silicon lattice. This layer has been shown to exhibit a denser SiO_2_ structure, resembling crystalline SiO_2_ polymorphs, as a result of strain during oxidation [46]. Its thickness is typically 1–2 nm [47,48]. Beyond this, a chemical transition layer is formed, characterized by the presence of silicon in intermediate oxidation states (Si^+1^, Si^+2^, Si^+3^). Depth profiling with X-ray photoelectron spectroscopy has shown that this chemical transition layer extends about 1 nm into the oxide and a few monolayers into the silicon [49].

The most prominent structural defect associated with the Si/SiO_2_ interface is the $P_{b}$ center ($\;\cdot \;\mathrm{Si}\equiv {\mathrm{Si}}^{3}$), consisting of a silicon atom bonded to three other Si atoms, leaving one unsaturated orbital [50], which is primarily located on the silicon side of the interface. Because the dangling orbital contains an unpaired spin, the $P_{b}$ center acts as an amphoteric carrier trap with multiple possible charge states: neutral ($P_{b}^{0}$), negative ($P_{b}^{-}$), or positive ($P_{b}^{+}$) [51].

The first lifetime component on the left side of Figure 6 increases as the positron drift from the oxide toward the interface becomes stronger. This lifetime component is attributed to defects located at the oxide–silicon interface, such as the $P_{b}$ center. In contrast, the shortest lifetime on the right side of Figure 6 is attributed to a reduced bulk lifetime of 185 ps. This value is shorter than both the lowest lifetime component observed in the oxide (excluding para-positronium) and the bulk lifetime of silicon. This can only be explained by positron trapping at the interface, which effectively reduces the measured bulk lifetime of silicon [25,52] from its intrinsic value of 221 ps [28]. If a defect-related component with a similar lifetime and intensity were present on the silicon side of the interface, it should be detectable in the lifetime spectrum.

The absence of detectable $P_{b}$-like lifetime components during drift from the silicon side may be attributed to the charge state of these centers [51]. The key difference between the two drift directions arises from the electronic configuration at the interface. During drift from the oxide side, the MOS capacitor operates in inversion mode. In this regime, free electrons accumulate at the interface, causing the $P_{b}$ centers to become negatively charged ($P_{b}^{-}$). This negative charge enhances positron trapping through Coulomb attraction. In contrast, during drift from the silicon side, the device operates in accumulation mode. Hole accumulation leads to positively charged $P_{b}^{+}$ centers, which repel positrons and thus suppress positron trapping at the interface.

Another observation in the oxide–interface drift regime is the presence of a void-like defect with a lifetime of $\tau_{2,\mathrm{ox}}=445\left(11\right)\;ps$. This lifetime component shows an abrupt change at low gate voltages and then remains nearly constant at higher voltages. In contrast, its intensity increases continuously with increasing drift strength, rising from $I_{2}=52\%$ to 61%. This increase occurs over the entire gate-voltage range, suggesting that a higher drift velocity leads to a larger fraction of positrons annihilating in this quantum state. This behavior is consistent with a reduction in positronium formation caused by charge separation under a strong electric field. As a result, more free positrons are driven toward the SiO_2_ interface, where the oxide microstructure appears to be dominated by such void-like defects.

When positrons are drifted from the silicon side (Figure 6, right column), a similar void-like component is observed with a lifetime of $\tau_{2,\mathrm{si}}=465\left(2\right)\;ps$. Density Functional Theory calculations show that this lifetime corresponds to large vacancy clusters in silicon containing more than 14 vacancies [53]. The observed lifetime difference of $\Delta \tau_{2}=20\left(1\right)\;ps$ between the two drift directions suggests variations in the local electronic environment at the annihilation sites, depending on whether positrons annihilate on the oxide or silicon side of the interface. On the oxide side, the higher oxygen concentration likely modifies the electronic structure of the voids, possibly through oxygen decoration of the vacancy-cluster walls, as proposed in [10]. The higher intensity of this component on the oxide side, approximately 15%, is further attributed to such clusters being intrinsic defects in SiO_2_. In contrast, defect-free silicon contains fewer such trapping sites, making positron trapping in these defects less efficient on the silicon side.

The results demonstrate that the SiO_2_/Si interface exhibits distinct electronic and microstructural characteristics depending on the direction of positron approach. The observed differences in lifetime components highlight the intrinsic asymmetry of the interface, consistent with earlier reports of structural disparities between the oxide and silicon sides [46]. Further insight into the interface structure and positron drift transport could be gained by extending such experiments to different oxide types (e.g., dry thermally grown, chemically deposited) with varying thicknesses. Equally important is the systematic variation of silicon properties such as doping concentration, doping type, and lattice orientation.

## 4. Conclusions

Characterizing vacancy-type defects in ultra-thin embedded layers and at buried interfaces is challenging when using conventional low-energy beam-based positron annihilation spectroscopy due to their broad implantation profiles. This work presents a proof-of-concept measurement demonstrating how the signal in positron annihilation lifetime experiments on such ultra-thin layers or buried interfaces can be enhanced by drifting positrons into the desired regions using externally applied electric fields.

Our study demonstrates that the electric field directly influences positron annihilation characteristics in the substrate. Under inversion conditions, the electric field near the interface acts as a diffusion barrier that pulls positrons away from the interface and transports them deeper into the substrate. During accumulation, a gate-voltage-dependent change in the annihilation properties is also observed, deviating from those expected for defect-free silicon. This behavior indicates that positrons are transported toward the SiO_2_/Si interface via a drift–diffusion mechanism. The annihilation characteristics at the interface are clearly distinct from those of the bare silicon surface.

By comparing positron drift from the silicon side with drift from the oxide side, a more detailed picture of the microstructure of the SiO_2_/Si interface can be obtained. The observed differences in annihilation characteristics for oxide-drifted and silicon-drifted positrons indicate that the defects on the silicon side of the interface differ from those on the SiO_2_ side. A possible explanation is charge-state switching of electronically active defects, such as $P_{b}$ centers, combined with chemical variations across the interface [46,51].

In conclusion, this study demonstrates that drift-assisted positron annihilation lifetime spectroscopy can be a powerful and effective technique for characterizing defects at buried interfaces in semiconductors and semiconductor–insulator junctions. By actively controlling positron transport with an electric field, this approach enables interface-sensitive defect identification that is not accessible with conventional methods. The methodology shows strong potential for the systematic investigation of defect landscapes in layered material systems for thin-film transistor technologies beyond silicon, such as HfO_2_. However, the general applicability of the method to such systems, as well as to advanced semiconductor power applications, has yet to be established and will be addressed in future studies.

Furthermore, we emphasize that although this work focuses primarily on the thin-film transistor-relevant structures, the methodology is equally applicable to the investigation of electronically active defects in other semiconductor devices, such as diodes [54] or piezoelectric systems [55]. 

## Figures and Tables

**Table 1:** Summary of the results of the positron lifetime analysis for the three-component decomposition. The same initial fit parameters were used for all lifetime spectra. The uncertainty of the gate voltage is ±0.5 V.

$U_{g}$ [V]	$\tau_{1}$ [ps]	$I_{1}$ [%]	$\tau_{2}$ [ps]	$I_{2}$ [%]	$\tau_{3}$ [ps]	$I_{3}$ [%]
−30	185(1)	52(1)	465(2)	46(1)	1270(16)	2.1(1)
−17	184(2)	51(1)	461(2)	46(1)	1274(12)	2.8(1)
0	186(1)	58(1)	476(3)	38(1)	1363(9)	4.1(1)
1	230(1)	93(1)	484(32)	3.8(5)	1386(10)	3.6(1)
19	233(1)	94(1)	544(29)	3.6(3)	1404(20)	1.9(1)
30	232(1)	95(1)	543(24)	4.0(3)	1422(26)	1.3(1)

## Data Availability

The data presented in this study are available on request from the corresponding author due to planned follow-up research.
